# Development of the aboral domain in *Nematostella* requires *β-catenin* and the opposing activities of *Six3/6* and *Frizzled5/8*

**DOI:** 10.1242/dev.120931

**Published:** 2016-05-15

**Authors:** Lucas Leclère, Markus Bause, Chiara Sinigaglia, Julia Steger, Fabian Rentzsch

**Affiliations:** 1 Sars Centre for Marine Molecular Biology, University of Bergen, Thormøhlensgt 55, Bergen 5008, Norway; 2 Sorbonne Universités, UPMC Univ Paris 06, CNRS, Laboratoire de Biologie du Développement de Villefranche-sur-mer (LBDV), 181 chemin du Lazaret, Villefranche-sur-mer 06230, France

**Keywords:** Anterior posterior axis, Apical organ, Axis formation, Cnidaria, Wnt signaling

## Abstract

The development of the oral pole in cnidarians and the posterior pole in bilaterians is regulated by canonical Wnt signaling, whereas a set of transcription factors, including Six3/6 and FoxQ2, controls aboral development in cnidarians and anterior identity in bilaterians. However, it is poorly understood how these two patterning systems are initially set up in order to generate correct patterning along the primary body axis. Investigating the early steps of aboral pole formation in the sea anemone *Nematostella vectensis*, we found that, at blastula stage, oral genes are expressed before aboral genes and that Nvβ-catenin regulates both oral and aboral development. In the oral hemisphere, Nvβ-catenin specifies all subdomains except the oral-most, *NvSnailA*-expressing domain, which is expanded upon *Nvβ-catenin* knockdown. In addition, Nvβ-catenin establishes the aboral patterning system by promoting the expression of *NvSix3/6* at the aboral pole and suppressing the Wnt receptor *NvFrizzled5/8* at the oral pole. *NvFrizzled5/8* expression thereby gets restricted to the aboral domain. At gastrula stage, *NvSix3/6* and *NvFrizzled5/8* are both expressed in the aboral domain, but they have opposing activities, with *NvSix3/6* maintaining and *NvFrizzled5/8* restricting the size of the aboral domain. At planula stage, *NvFrizzled5/8* is required for patterning within the aboral domain and for regulating the size of the apical organ by modulation of a previously characterized FGF feedback loop. Our findings suggest conserved roles for *Six3/6* and *Frizzled5/8* in aboral/anterior development and reveal key functions for *Nvβ-catenin* in the patterning of the entire oral-aboral axis of *Nematostella*.

## Abstract (precis)

**Summary:** Wnt signalling and the Six3/6 transcription factors play conserved functions in primary axis patterning in bilateria, controlling aboral/anterior identity in *Nematostella.*

## INTRODUCTION

The establishment of distinct territories along the anterior-posterior body axis is a key step in animal embryogenesis. Conserved patterning molecules specify the anterior and posterior territories of bilaterians. We address here how these patterning systems are established and how their interaction can pattern the main body axis of the cnidarian *Nematostella vectensis*, in order to gain further insights into the mechanisms that control axial patterning and their evolution.

The key determinant for the posterior pole of bilaterians is canonical Wnt/β-catenin signaling. In this pathway, binding of secreted Wnt ligands to Frizzled transmembrane receptors leads to the inactivation of a ‘destruction’ complex that includes Axin, APC and GSK3. In the absence of a Wnt signal, this complex marks cytoplasmic β-catenin for proteasome-mediated degradation whereas inactivation of the complex allows β-catenin to enter the nucleus and regulate gene expression together with the transcription factor TCF (Croce and McClay, 2006; MacDonald et al., 2009). In bilaterians, β-catenin signaling can have several roles during early development. Nuclearization of β-catenin during cleavage stages is an early sign of embryonic polarity; it is often restricted to the vegetal hemisphere and it is essential for specifying the site of gastrulation and for endoderm formation in a broad range of species (Darras et al., 2011; Henry et al., 2008; Lhomond et al., 2012; McIntyre et al., 2013; Petersen and Reddien, 2009; Range et al., 2013; Yaguchi et al., 2008, 2006). In vertebrates, β-catenin is also required for the formation of embryonic organizers, which regulate both anterior-posterior and dorsal-ventral patterning (De Robertis and Kuroda, 2004; Niehrs, 2004). Subsequently, β-catenin can have a direct role in the patterning of the anterior-posterior axis by transducing Wnt signals emanating from the posterior region (Kiecker and Niehrs, 2001; Niehrs, 2004). In this regard, inhibition of β-catenin function leads to a reduction of the expression of posterior marker genes and a concomitant expansion of anterior markers (Bellipanni et al., 2006; Darras et al., 2011; Fu et al., 2012; Heasman et al., 1994; Henry et al., 2010, 2008; Logan et al., 1999; Marlow et al., 2014; Wikramanayake et al., 1998). Accordingly, protection from posteriorizing Wnt/β-catenin signaling is thought to be necessary for the development of the anterior ectoderm.

An important patterning gene for the anterior domain is the homeodomain transcription factor *Six3/6*, which is expressed in the anterior-most part of various bilaterians, as shown in lophotrochozoans, ecdysozoans and deuterostomes (Kozmik et al., 2007; Lowe et al., 2003; Oliver et al., 1995; Posnien et al., 2009; Poustka et al., 2007; Seo et al., 1998; Steinmetz et al., 2010; Zhou et al., 2000). Functional studies demonstrated that inhibition or mutation of *Six3/6* leads to severe deficiencies in anterior development of the beetle *Tribolium castaneum* (Posnien et al., 2011) and the sea urchin *Strongylocentrotus purpuratus* (Wei et al., 2009), as well as truncation of the anterior brain in vertebrates (Carl et al., 2002; Lagutin et al., 2003). Other genes that are consistently expressed at the anterior pole of bilaterian embryos include the transcription factors *FoxQ2*, *Pax6*, *Nkx2.1* and *Fez*, and the signaling molecules *Frizzled5/8* and *sFRP1* (summarized by Petersen and Reddien, 2009; Sinigaglia et al., 2013).

As the sister group of bilaterians, cnidarians (corals, sea anemones and jellyfish) hold an informative phylogenetic position for understanding the evolution of body patterning (Dunn et al., 2014; Holstein et al., 2011; Telford et al., 2015). Cnidarians lack the mesodermal germ layer and they display only one externally visible body axis, termed the oral-aboral axis. In contrast to bilaterians, gastrulation and endoderm formation occur in the territory derived from the animal hemisphere of the oocyte. As in bilaterians, Wnt/β-catenin signaling is the key determinant of the site of gastrulation and endoderm formation, and it promotes oral identity, as shown in the hydrozoans *Clytia hemisphaerica* and *Hydractinia echinata* (Lapebie et al., 2014; Momose et al., 2008; Momose and Houliston, 2007; Plickert et al., 2006) and in the anthozoan *Nematostella vectensis* (Wikramanayake et al., 2003; Lee et al., 2007; Kumburegama et al., 2011; Rottinger et al., 2012).

*Nematostella* gastrulates by invagination and develops into a ciliated, free-swimming planula before becoming a sessile polyp (Kraus and Technau, 2006; Magie et al., 2007). The Wnt pathway component NvDishevelled is localized to the animal pole from the oocyte until gastrula stage (Lee et al., 2007), Nvβ-catenin is preferentially stabilized in the animal/oral region of the blastula (Wikramanayake et al., 2003) and *Nematostella* Wnt ligands are expressed in staggered domains exclusively in the oral half of the embryo from blastula stage onwards (Kusserow et al., 2005). *Nvβ-catenin* (*Nvβ*-*cat*) has been shown to be required for proper endoderm formation (Kumburegama et al., 2011; Lee et al., 2007; Wikramanayake et al., 2003) and inhibition of the β-catenin co-factor *NvTcf* suggested that canonical Wnt signaling affects ectodermal patterning by restricting the size of the aboral domain (Rottinger et al., 2012).

The aboral pole in *Nematostella* expresses orthologs of the bilaterian anterior patterning genes *Six3/*6 and *FoxQ2* and gene knockdown experiments have identified *NvSix3/6* as a key regulator for the development of a broad aboral territory (Sinigaglia et al., 2013). Whereas knockdown of *NvSix3/6* leads to an expansion of *NvWnt2* expression towards the aboral pole (Sinigaglia et al., 2013), overactivation of Wnt/β-catenin signaling by azakenpaullone treatment leads to the repression of *NvSix3/6* and *NvFoxQ2a* expression (Marlow et al., 2013; Sinigaglia et al., 2013). These findings support a model in which *NvSix3/6* and Wnt/β-catenin signaling counteract each other in the demarcation of oral and aboral territories at gastrula and planula stage.

In this article, we investigate the establishment of the aboral domain in *Nematostella*. We show that rather than promoting oral at the expense of aboral domain development, *Nvβ-catenin* is required for the formation of both oral and aboral territories. *Nvβ-catenin* regulates the development of the aboral territory by directly or indirectly activating *NvSix3/6* expression at the aboral pole and suppressing the Wnt receptor *NvFz5/8* at the oral pole. We further show that at gastrula stage the opposing activities of *NvSix3/6* and *NvFz5/8* maintain the size of the aboral domain and that at planula stage an interaction between *NvFz5/8* and fibroblast growth factor (FGF) signaling refines the patterning of this domain.

## RESULTS

### *NvSix3/6* controls but does not initiate aboral domain development

**Fig. 1.**
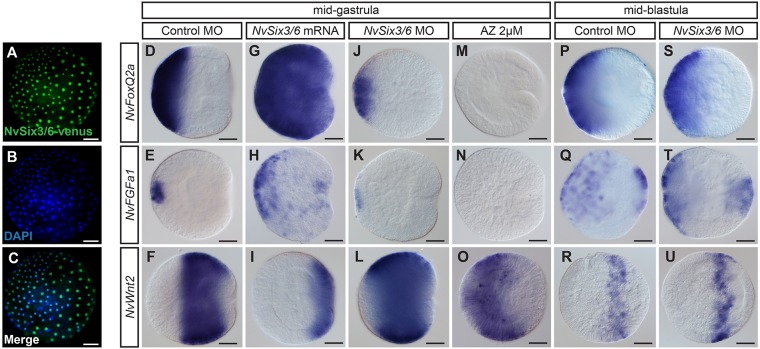
***NvSix3/6* controls but does not initiate aboral domain development.** (A-C) Nuclear staining of NvSix3/6-Venus fusion protein (green) and DAPI (blue) at blastula stage. (D-U) Lateral views (aboral pole to the left) of *in situ* hybridizations showing expression patterns of *NvFoxQ2a*, *NvFGFa1* and *NvWnt2* at mid-gastrula (26 hpf; D-O) or mid-blastula stage (14 hpf; P-U) embryos. Injected morpholinos, mRNA or AZ treatment (2 µM, 2-26 hpf) are indicated above the panels, probes on the left. Scale bars: 50 µm.

In *Nematostella*, knockdown of *NvSix3/6* leads to loss of expression of the aboral markers *NvFGFa1*, *NvFGFa2* and *NvFoxQ2a* and expansion of the belt-like central ectodermal marker *NvWnt2* towards the aboral pole (Sinigaglia et al., 2013; Fig. 1J-L). Here, we tested whether *NvSix3/6* is sufficient to induce the ectopic expression of aboral markers. Injection of *NvSix3/6-venus* mRNA led to an expansion of the aboral marker genes *NvFoxQ2a* and *NvFGFa1*, and to a corresponding shift towards the oral pole of the *NvWnt2* domain (Fig. 1A-I). Together with the suppression of aboral markers upon pharmacological Wnt/β-catenin overactivation (Fig. 1M-O; Marlow et al., 2013; Rottinger et al., 2012; Sinigaglia et al., 2013), this observation supports the hypothesis that the embryo is patterned by the opposing activities of a *NvSix3/6*-controlled aboral module and an oral module controlled by canonical Wnt signaling, and that mutual suppression is an important function of these two modules.

Next, we analyzed the effects of *NvSix3/6* MO injection at mid-blastula stage and found that the size of the aboral domain was not significantly affected, as seen by the expression of *NvFoxQ2a*, *NvFGFa1*, *NvWnt2* (Fig. 1P-U) and *NvFkh* (Fig. S1). This indicates that aboral identity is initially defined independently of *NvSix3/6* activity.

### The onset of expression of oral markers precedes that of aboral markers

In order to understand how the oral and aboral domains are initially established, we first determined the onset of localized expression for a set of oral and aboral marker genes. The expression of oral markers consistently preceded that of aboral markers by 1-2 h [10 hours post-fertilization (hpf) versus 11 hpf at 21°C; Fig. S2]. At mid-blastula stage (11-12 hpf), the prospective oral territory appears to be already subdivided in staggered domains, with *NvWntA*, *NvSnailA* and *NvFGFa1* co-expressed in the oral-most subdomain, surrounded by a central ring of expression of *NvFkh* and *NvFoxB*, and *NvWnt2* expressed in an outer-most ring; at the same time, *NvSix3/6*, *NvFoxQ2a* and *NvFGFa1* were co-expressed in the aboral half (Fig. S2). Interestingly, *NvFGFa1* oral expression appeared 1-2 h before aboral expression; as determined by one-color double *in situ* hybridization for *NvFGFa1* and *NvWnt2* at 10 hpf, which showed staining only at one pole (data not shown). The same four domains were also described at late blastula stage (Rottinger et al., 2012), with the exceptions of *NvWntA*, which is at this stage co-expressed with *NvFkh* and *NvFoxB* in the central ring, and *NvFGFa1*, which is no longer expressed in the oral half.

### β-catenin activity is required for the expression of both oral and aboral marker genes

**Fig. 2.**
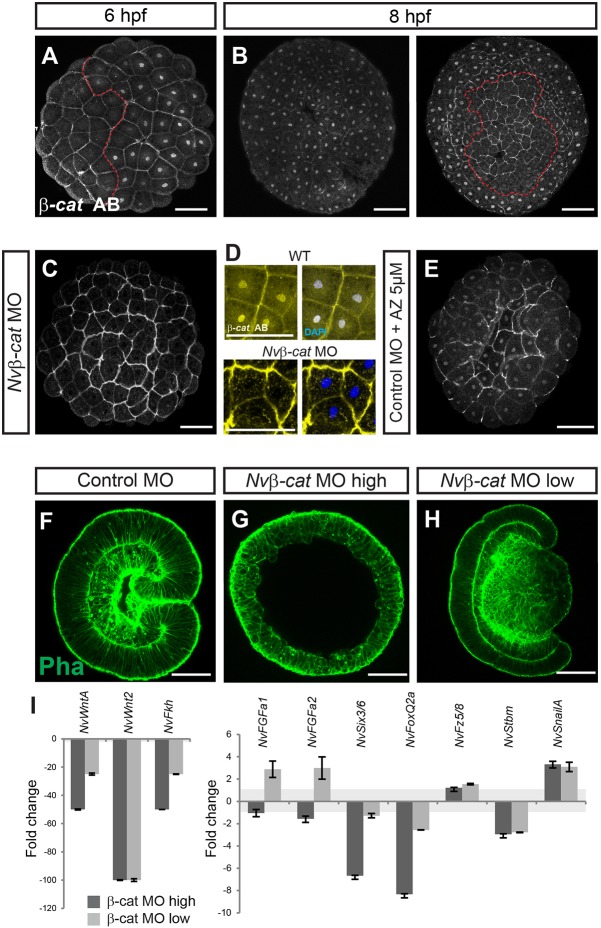
**Nvβ-catenin is necessary for gastrulation and for transcription of both oral and aboral marker genes.** (A-E) Early blastulae labeled with anti-mouse β-catenin antibody (white in A-C,E; yellow in D). In D, DAPI is blue and colocalization is white. Treatments are indicated on the left side. Dashed red line in A,B delimits the area of weak nuclear β-catenin antibody staining*.* The two images in B represent maximum projections of the two halves of the same embryo. *Nvβ-cat* MO (500 µM) reduces and AZ treatment enhances labeling. Note that the embryo in A is 6 hpf whereas the others are 8 hpf. MO injection causes a developmental delay (C,E) compared with wild-type (WT) embryos (A,B). (F-H) Phalloidin (green) staining of control MO (F) and *Nvβ-cat* MO (G, 500 μM, H, 100 μM) injected embryos at 26 hpf. (I) RT-qPCR of *Nvβ-cat* MO-injected embryos (28 hpf) at a concentration of 500 µM (dark gray) and 100 µM (light gray) compared with the control (control MO, 500 µM). Fold changes of the relative expression levels are shown; values between −1 and +1 mean no change (highlighted in light gray). Error bars represent the s.d. of three (500 µM) or two (100 µM) biological replicates in two technical replicates each. Scale bars: 50 µm.

To investigate the role of the Wnt/β-catenin pathway in defining different territories along the oral-aboral axis, we employed two different antisense morpholinos (MO1 and MO2) targeting the key intracellular effector of this pathway, *Nvβ-cat*. Injection of either morpholino resulted in the same morphological phenotype and the same changes in marker gene expression, whereas the corresponding mismatch morpholinos had no effect (see below and Fig. S3). To assess the reduction of Nvβ-cat protein upon morpholino injection, we employed an antibody against mouse β-catenin. In immunofluorescence preparations, this antibody labeled nuclei in an area encompassing ∼70% of the embryo, as well as the cell cortex and, weakly, the cytoplasm in the whole embryo (Fig. 2A,B), as expected for β-catenin. This staining was detectable until early blastula stage (8 hpf at 21°C; Fig. 2B). After this stage (corresponding to the onset of expression for the earliest oral markers), the cytoplasm and the nucleus were uniformly stained, with slightly stronger signal at the cell cortex (data not shown). We suspect that the uniform intracellular staining is due to non-specific labeling at these later stages; it precluded the analysis of potential differences in the levels of nuclear Nvβ-catenin. In embryos (8 hpf) injected with 500 µM of *Nvβ-cat* MO1 or MO2 no nuclear signal was detectable and the staining at the cell cortex was strongly reduced (Fig. 2C,D; Fig. S3). By contrast, treatment with the GSK3 inhibitor 1-azakenpaullone (AZ), which reduces the degradation of β-catenin (Kunick et al., 2004), enhanced the signal in all nuclei (Fig. 2E), suggesting that this antibody does indeed detect *Nematostella* β-catenin. Embryos injected with a high concentration of *Nvβ-cat* MO (500 µM; MO2 was used here and for all further experiments) developed into blastula-like epithelial spheres, but did not show any morphological sign of body polarity and did not gastrulate (Fig. 2F,G). This phenotype is similar to that obtained after injection of *Cadherin* mRNA, which prevents nuclearization of β-catenin (Logan et al., 1999; Wikramanayake et al., 2003), but more severe than those described for the injection of either *Axin* mRNA (Kumburegama et al., 2011) or a dominant-negative *NvTcf* construct (Rottinger et al., 2012). Upon injection of a lower concentration of *Nvβ-cat* MO (100 µM), the embryos developed an endoderm-like tissue consisting of relatively big cells, and initiated gastrulation-like tissue invagination but failed to complete this process, resulting in a partially invaginated endoderm that frequently bulged out towards the oral pole (Fig. 2H). Reverse transcription quantitative PCR (RT-qPCR) at a time point when control MO-injected animals had reached the mid-gastrula stage showed that the high dose of *Nvβ-cat* MO (500 µM) reduced the expression of the oral and mid-body markers *NvWntA*, *NvFkh* (both >40-fold) and *NvWnt2* (∼100-fold; Fig. 2I) (Fritzenwanker et al., 2004; Kusserow et al., 2005; Martindale et al., 2004), an effect more than tenfold stronger than that observed upon injection of dominant-negative *NvTcf* construct (Rottinger et al., 2012).

**Fig. 3.**
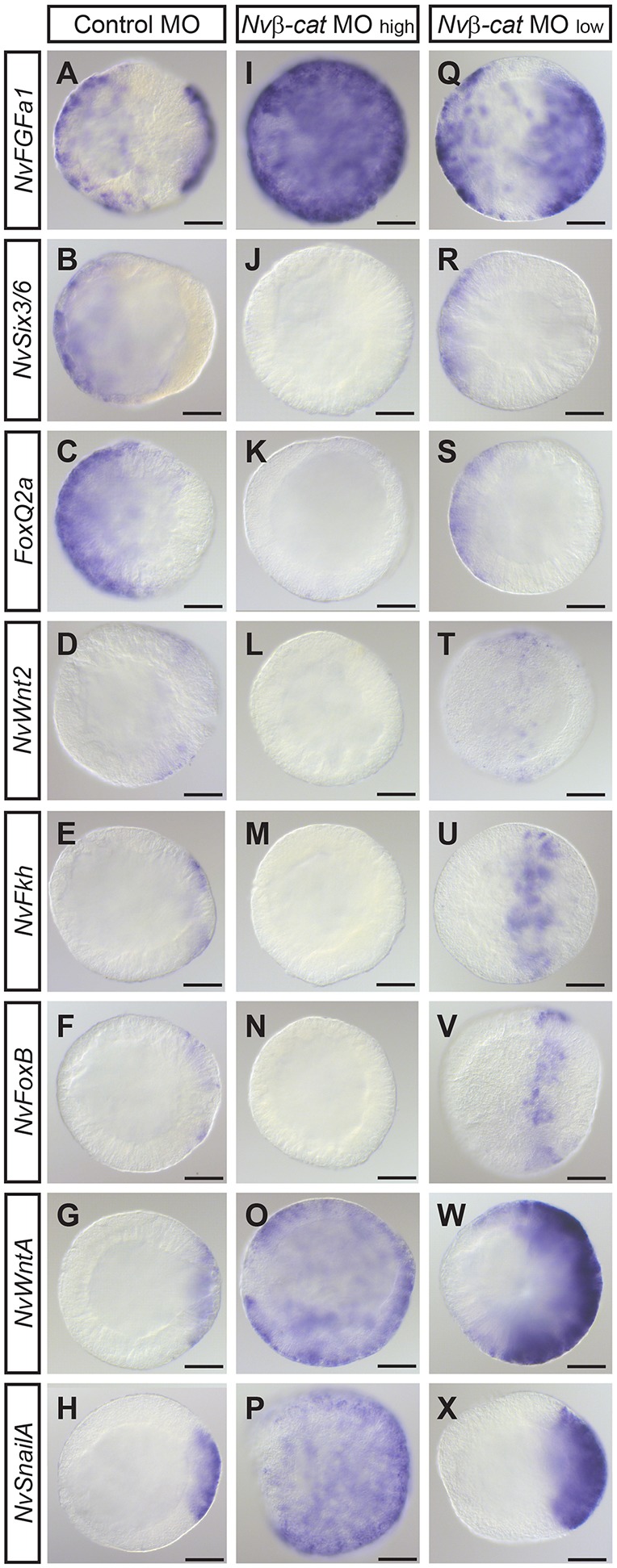
***Nvβ-cat* is required for the establishment of oral and aboral patterning systems.** (A-X) *In situ* hybridizations at mid-blastula stage (14 hpf). Probes are indicated on the left, injections indicated above. Lateral views, the future aboral pole to the left, assuming continuity of the expression of marker genes at gastrula stage, when the blastopore becomes visible. The presence of oral and aboral expression domains for *NvFGFa1* makes the orientation in Q unreliable. Note the shift of *NvFkh* (U) and *NvFoxB* (V) away from the oral pole in low dose *Nv*β*-cat* MO-injected blastulae. Expression patterns shown in U,V were observed in ∼50% of the embryos; the remaining 50% showed no staining (*NvFkh*: 16/34; *NvFoxB*: 13/25). Scale bars: 50 μm.

To determine the function of *Nvβ-catenin* in axial patterning, we analyzed the effects of injection of *Nvβ-cat* MO on the expression of several marker genes. The selected time point was mid-blastula stage (14 hpf), shortly after localized expression of both oral and aboral markers was observed in control animals. At the high dose of *Nvβ-cat* MO, expression of the oral markers *NvWnt2*, *NvFkh* and *NvFoxB*, but also of the aboral markers *NvSix3/6* and *NvFoxQ2a*, was strongly reduced or lost (Fig. 3B-F,J-N). Surprisingly, markers of the oral-most domain, *NvWntA*, *NvSnailA* and *NvFGFa1* (which at this stage has an aboral and an oral expression domain) were ubiquitously expressed in *Nvβ-cat* morphants at blastula stage (Fig. 3A,G,H,I,O,P). When injected at the lower concentration, the *Nv*β*-cat* morpholino affected the expression of all markers in a similar, albeit weaker, manner, with the boundaries of the expression domains all shifted towards the aboral pole (Fig. 3Q-X). These results suggest that reducing the levels of Nvβ-cat can affect regional identity along the oral-aboral axis.

**Fig. 4.**
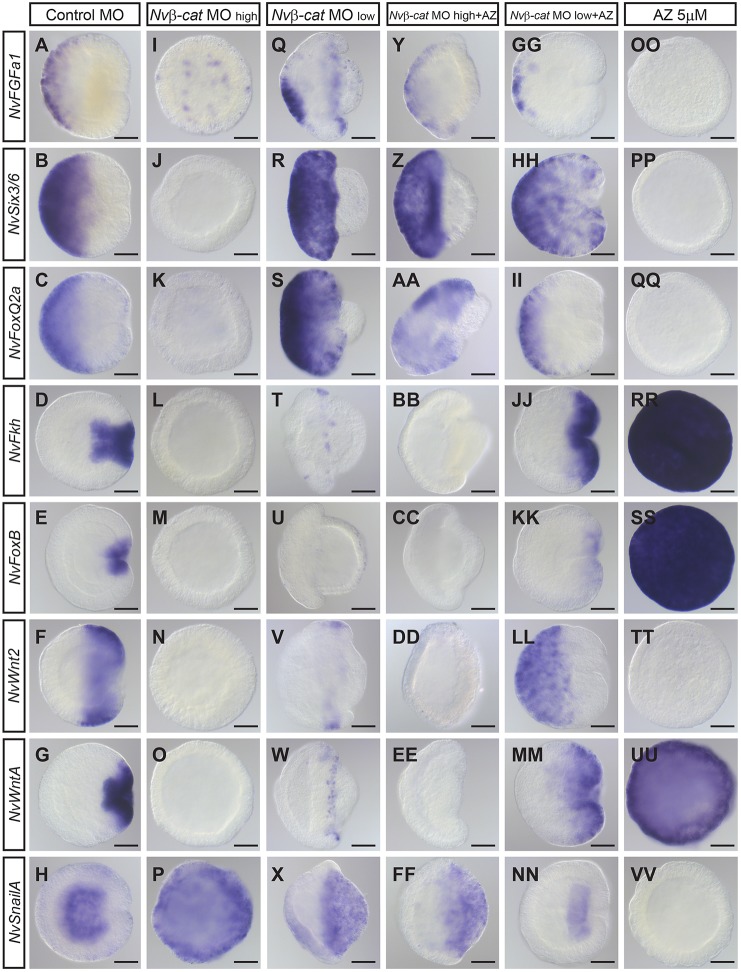
**Loss of polarity upon *Nvβ-cat* knockdown is partially rescued by AZ treatment.** (A-VV) Lateral views (aboral pole to the left) of *in situ* hybridizations at mid-gastrula stage (26 hpf). Probes are indicated on the left, treatments/injections indicated above. *Nvβ-cat* MO high: 500 µM; *Nvβ-cat* MO low: 100 µM; AZ: 5 µM, 4-26 hpf. Animals injected with high *Nvβ-cat* MO dose and treated with AZ (Y-FF) resemble those injected with low *Nvβ-cat* MO dose (Q-X); animals injected with low *Nvβ-cat* MO dose and treated with AZ (GG-NN) resemble those injected with control MO (A-H), except for *NvWnt2* (F,LL). Scale bars: 50 μm.

We next tested whether the effects of Nvβ-cat reduction persist at a later time point, corresponding to gastrulation in control animals (26 hpf). At this stage, *NvFkh* and *NvFoxB* are expressed in the pharynx; *NvWntA* is no longer co-expressed with *NvSnailA*, but instead localizes around the blastopore, partially overlapping with *NvFkh*; and the aboral expression domain of *NvFGFa1* is narrower than that of *NvSix3/6* and *NvFoxQ2a* (Fig. 4A-H). Similar to blastula stage, expression of the oral markers *NvWnt2*, *NvFkh* and *NvFoxB*, and of the aboral markers *NvSix3/6* and *NvFoxQ2a* was strongly reduced or abolished upon injection of the high concentration of *Nvβ-cat* MO (Fig. 4J-N), whereas the expression of *NvSnailA* was expanded throughout the body column (Fig. 4H,P). A notable difference to the situation at blastula stage was the expression of *NvWntA*, which at gastrula stage was no longer ubiquitously expressed but instead was strongly reduced (Fig. 4G,O). The aboral marker *NvFGFa1* was at gastrula stage expressed in sparse patches throughout the body column (Fig. 4I). Similar to the observations at blastula stage, injection of the low *Nvβ-cat* MO concentration affected the expression of *NvWnt2*, *NvWntA*, *NvFkh* and *NvFoxB* to a lesser extent (Fig. 4T-W); *NvSnailA* expression was strongest in the oral endoderm-like tissue (Fig. 4X). The expression of *NvSix3/6* and *NvFoxQ2a* expanded throughout the ectodermal tissue in embryos injected with the lower *Nvβ-cat* MO concentration (Fig. 4R,S), in contrast to the strong reduction upon injection of the high MO concentration. *NvFGFa1* was expressed in patches (as at the high MO concentration) and in an aboral domain (Fig. 4Q) and it was upregulated in RT-qPCR analysis (Fig. 2I).

To confirm that the changes in gene expression are indeed due to the reduced levels of Nvβ-cat, we re-activated β-catenin signaling in *Nvβ-cat* MO-injected animals by AZ treatment. We reasoned that the *Nvβ-cat* MO does not completely eliminate Nvβ-cat protein and that a low amount of residual protein could be stabilized by AZ, without affecting potential differences in the levels of Nvβ-cat along the oral-aboral axis. Remarkably, animals injected with the high dose of *Nvβ-cat* MO and treated with 5 μM AZ from 4 hpf on (which by itself caused strong oralization, Fig. 4OO-VV) displayed a morphology and patterns of gene expression that were similar to animals that were injected with the low dose of *Nvβ-cat* MO alone (Fig. 4Y-FF). Moreover, AZ treatment of animals injected with the low dose of *Nvβ-cat* MO resulted in near-wild-type morphology and expression of most marker genes in domains similar to control animals (Fig. 4GG-KK,MM,NN), with the exception of *NvWnt2*, which was expressed in the aboral domain (Fig. 4LL).

Taken together, these results show that Nvβ-cat acts as a positive regulator of several oral and aboral genes already at the blastula stage, when localized expression of these markers first becomes visible. Interestingly, markers of the oral-most territory, the central domain (Rottinger et al., 2012), which presumably gives rise to the endodermal plate, are upregulated upon knockdown of *Nvβ-cat*, suggesting the existence of additional regulators of oral gene expression.

### The Wnt receptor *NvFz5/8* is expressed at the aboral pole

Next, we addressed how the relative extensions of the oral and aboral territories are regulated after the initial set-up of the body pattern. Interestingly, the Wnt receptor *NvFz5/8* is expressed in the aboral domain of *Nematostella* at early gastrula stage (Kumburegama et al., 2011; Sinigaglia et al., 2015), thus providing a candidate for a crosstalk between oral Wnt signaling activity and the aboral domain.

**Fig. 5.**
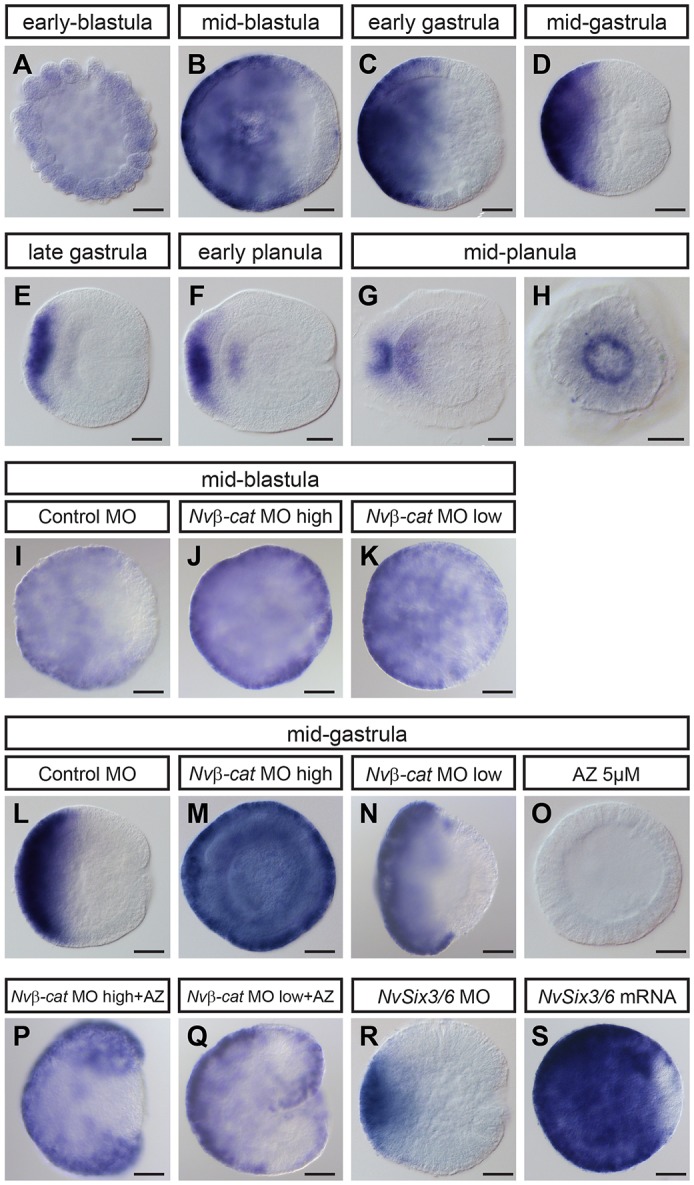
**Aboral domain expression of *NvFz5/8* requires oral suppression by *Nvβ-cat* and maintenance by *NvSix3/6*.** (A-S) *NvFz5/8 in situ* hybridizations of uninjected animals (A-H) and animals treated/injected as indicated (I-S). All images are lateral views, except H which is an aboral view. Stages are indicated above. *Nvβ-cat* MO high: 500 µM; *Nvβ-cat* MO low: 100 µM; AZ: 5 µM, 4-26 hpf. Scale bars: 50 µm.

*NvFz5/8* is ubiquitously expressed during early cleavage stages (Fig. 5A and data not shown), indicating its maternal origin. Its expression becomes progressively restricted to the future aboral domain during blastula stage, starting from 11 hpf (Fig. 5B; Fig. S2). From late blastula until mid-gastrula stage the *NvFz5/8* expression pattern is highly similar to that of *NvSix3/6* (Fig. 5C,D), although there might still be slight differences in their extension towards the oral pole. Between late gastrula and early planula (48 hpf), *NvFz5/8* expression becomes gradually more restricted to the ectoderm of the aboral pole, with a strong expression in the apical organ and weaker expression in the adjacent ectoderm (Fig. 5E,F). At the mid-planula stage (72 hpf), the aboral expression domain forms a ring around the apical pole (Fig. 5H). At planula stages, an additional endodermal expression domain, just beneath the apical organ region, becomes detectable (Fig. 5F,G).

### *NvFz5/8* expression is controlled by *Nvβ-cat* and *NvSix3/6*

In order to investigate how the expression of *NvFz5/8* is regulated, we perturbed the two key axial patterning factors, *Nvβ-cat* and *NvSix3/6*. Injection of *Nvβ-cat* MO resulted in ubiquitous *NvFz5/8* expression at blastula and gastrula stage, thus resembling the early blastula stage of control embryos (Fig. 5I,J,L,M). The overall levels of *NvFz5/8* transcripts were, however, not affected, as shown by RT-qPCR (Fig. 2I). Conversely, stabilization of Nvβ-cat by AZ completely suppressed *NvFz5/8* expression (Fig. 5O). This suggests that the restriction of *NvFz5/8* to the aboral domain depends on a specific level of β-catenin activity. This was confirmed by low dose *Nvβ-cat* MO experiments at blastula and gastrula stage, showing asymmetric expression along the oral-aboral axis (Fig. 5K,N). Furthermore, AZ treatment restored the asymmetric expression of *NvFz5/8* caused by the *Nvβ-cat* MO (Fig. 5P,Q). Finally, knockdown and overactivation of *NvSix3/6* by injection of *NvSix3/6* MO and *NvSix3/6-venus* mRNA, respectively, showed that at gastrula, but not at blastula stage, the expression of *NvFz5/8* is positively regulated by *NvSix3/6* (Fig. 5R,S; Fig. S1).

In conclusion, regulation of *NvFz5/8* expression follows three steps: (1) it first requires an unknown maternal input; (2) it then becomes suppressed in the oral domain by relatively high Nvβ-cat activity; and (3) it becomes regulated by *NvSix3/6* after the aboral program is activated at late blastula stage.

### *NvFz5/8* limits the size of the aboral domain during gastrulation

**Fig. 6.**
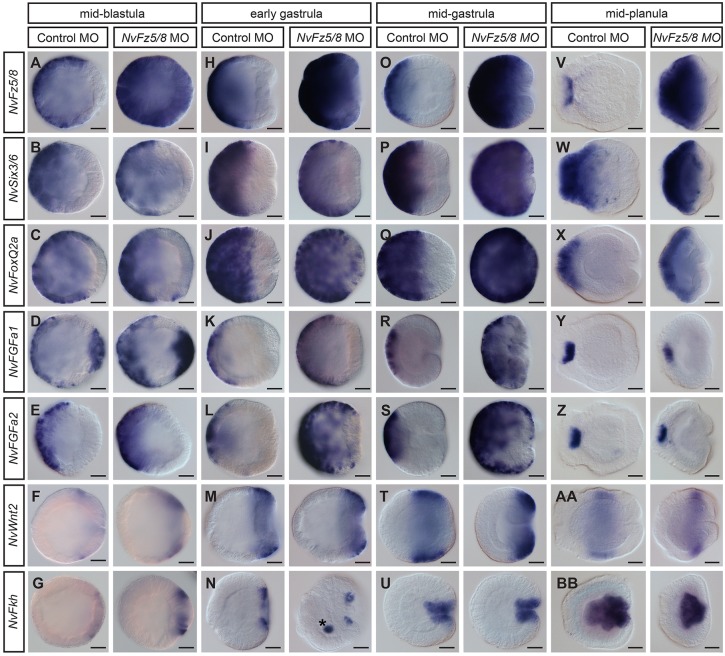
***NvFz5/8* limits the size of the aboral domain during gastrulation.** (A-BB) Lateral views (aboral pole to the left) of *in situ* hybridizations at the stages indicated above. Injections are also indicated above, probes on the left. The expansion of aboral marker gene expression and the reduction of oral markers are first visible at early gastrula stage and become more pronounced at mid-gastrula stage; see main text for more details. Asterisk in N indicates non-specific staining. Scale bars: 50 µm.

**Fig. 7.**
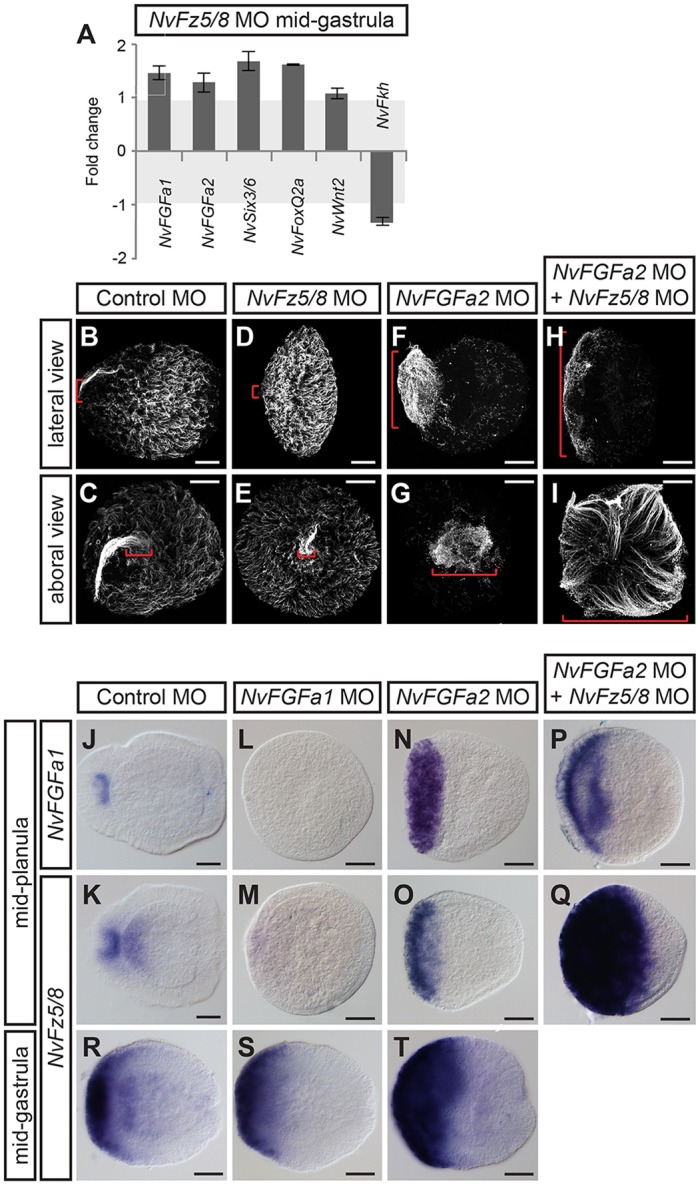
***NvFz5/8* regulates the size of the apical organ by controlling FGF activity in the aboral-most domain.** (A) RT-qPCR of *NvFz5/8* MO-injected embryos at mid-gastrula (26 hpf). Fold changes of the relative expression levels of the indicated genes are shown; values between −1 and +1 mean no change (highlighted in light gray). Error bars represent the s.d. of three biological replicates. (B-I) Lateral views (aboral pole to the left; B,D,F,H) and aboral views (C,E,G,I) of the apical ciliary tuft visualized by anti-acetylated tubulin antibody staining of mid-planulae. The injected MOs are indicated above. The red brackets highlight the size of the apical tuft in the different conditions. (J-T) Lateral views (aboral pole to the left) of *NvFGFa1* and *NvFz5/8 in situ* expression patterns in mid-planula (J-Q) and mid-gastrula (R-T) MO-injected embryos. The injected MOs and probes used are indicated above and on the left, respectively. Scale bars: 50 µm.

The aboral expression of *NvFz5/8* suggests it could play a role in the transduction of a Wnt signal originating from the oral domain, thus leading to *Nv*β-cat activity in the aboral domain. Injection of *NvFz5/8* antisense morpholino (see Fig. S4 for *NvFz5/8* MO control experiments) had no evident effect on aboral markers at blastula stages, except on its own expression (Fig. 6A-G), suggesting that it plays no major role in patterning at this stage. However, from early gastrula on, knockdown of *NvFz5/8* led to an expansion of all aboral markers analyzed, including *NvSix3/6* (Fig. 6H-L), whereas the mid-body and oral markers *NvWnt2* and *NvFkh* were only mildly affected (Fig. 6M,N). From mid-gastrula stage on, the expansion of aboral marker gene expression became more pronounced in *NvFz5/8* knockdown animals: *NvSix3/6* and *NvFoxQ2a* were clearly expanded towards the oral pole and, contrary to control mid-gastrula embryos, the expression domain of *NvWnt2* was shifted towards the oral pole (Fig. 6O-T). These results were confirmed by RT-qPCR showing a slight increase in the expression of all aboral markers (Fig. 7A). The corresponding mismatch MO did not have an effect on gene expression at mid-gastrula stage compared with the standard control MO (not shown). As an independent tool to manipulate the function of *NvFz5/8*, we injected mRNA for a truncated version of NvFz5/8 (encoding the extracellular and the first transmembrane domains fused to the open reading frame of the fluorescent protein Venus), which has been used as a dominant-negative construct in other organisms (Croce et al., 2006; Kim et al., 2002). Injection of this mRNA resulted in expanded expression of *NvSix3/6* and orally shifted expression of *NvWnt2* (Fig. S4). A developmental time course for the expression of the marker genes shows that the changes in gene expression do not correspond to a developmental delay (Fig. S5). Together, these data suggest that *NvFz5/8* negatively regulates *NvSix3/*6 and thereby limits the size of the aboral domain.

### *NvFz5/8* regulates the size of the apical organ by interacting with FGF signaling in the aboral domain

At mid-planula stage, the expression of *NvSix3/6* and *NvFoxQ2a*, and that of *NvFGFa1* and *NvFGFa2*, has segregated into separate domains, with *NvSix3/6* and *NvFoxQ2a* surrounding the expression of *NvFGFa1* and *NvFGFa2* in the apical organ (Fig. S6; Sinigaglia et al., 2013). At this stage, we could detect an expansion of *NvFz5/8*, *NvSix3/6* and *NvFoxQ2a* and a shift of the mid-body marker *NvWnt2* towards the oral pole in *NvFz5/8* MO-injected embryos. However, the expression of *NvFGFa1* and *NvFGFa2* was not affected at mid-planula stage (Fig. 6V-BB). *NvFz5/8* MO-injected planulae were also strikingly compressed along the oral-aboral axis (Fig. S7).

The differentiation of the apical organ occurs at planula stage and depends on FGF signaling, which acts through a feedback loop involving the activator *NvFGFa1* and the repressor *NvFGFa2* (Rentzsch et al., 2008). This feedback loop requires the input of *NvSix3/6* but becomes fully active only after gastrulation (Sinigaglia et al., 2013). We found here that *NvFz5/8* MO-injected planulae develop a slightly smaller apical organ (Fig. 7B-E).

As described above, in *NvFz5/8* MO-injected mid-planulae, *NvFGFa1* and *NvFGFa2* expression was restricted to the apical organ area as in control embryos (Fig. 6Y,Z), in contrast to the gastrula stages, when injection of *NvFz5/8* MO caused upregulation of these markers (Fig. 6K,L,R,S). We hypothesized that the reduction of the aboral-most domain between late gastrula and early planula stages could be due to the establishment of the self-regulating FGF feedback loop. In this feedback loop, *NvFGFa1* promotes the expression of *NvFGFa2*, which in turn represses the expression of *NvFGFa1* (Rentzsch et al., 2008). *NvFGFa1* represses *NvSix3/6* transcription, thus allowing the differentiation of the apical organ from an *NvFGFa1*/*NvFGFa2*-expressing and *NvSix3/6*-free spot within the aboral domain of the planula (Sinigaglia et al., 2013). In *NvFz5/8* MO-injected planulae, the expression pattern of *NvSix3/6* and *NvFoxQ2a* did indeed show a gap in the apical organ region (Fig. S6), albeit of reduced size compared with control MO-injected animals (also shown in Fig. S6). The expression patterns of *NvSix3/6*, *NvFGFa1*, *NvFGFa2* and *NvFz5/8* in control embryos suggests that at planula stage the expression of *NvFz5/8* is no longer under the control of *NvSix3/6*, but instead might depend on FGF signaling. Consistent with this hypothesis, injection of *NvFGFa1* MO, which produces planulae lacking an apical organ, suppressed the expression of *NvFz5/8* (Fig. 7J-M), whereas injection of *NvFGFa2* MO, which generates an expansion of the apical organ (Fig. 7F,G) (Rentzsch et al., 2008), caused an expansion of the ectodermal expression domain of both *NvFGFa1* and *NvFz5/8* (Fig. 7N,O). Double injection of *NvFGFa2* MO and *NvFz5/8* MO led to the formation of an even wider apical organ, covering approximately one third of the ectoderm (Fig. 7H,I) and a corresponding expansion of *NvFGFa1* and *NvFz5/8* expression domains (Fig. 7P,Q).

Finally, the change in the regulation of the expression of *NvFz5/8* between gastrula and planula stages was corroborated by analyzing these phenotypes at gastrula stage. In contrast to *NvSix3/6* MO (Fig. 5R), injection of *NvFGFa1* MO did not seem to affect the expression pattern of *NvFz5/8* at this stage, whereas injection of *NvFGFa2* MO caused a slight expansion of the *NvFz5/8* domain, as also seen for other aboral markers (Fig. 7R-T; Sinigaglia et al., 2013). These observations support the idea that *NvFz5/8* contributes to patterning within the aboral domain by interacting with the NvFGF signaling feedback loop.

## DISCUSSION

In the present study, we show that activity of Nvβ-catenin is required for the formation of both the oral and the aboral domain in *Nematostella* embryos. Nvβ-catenin establishes the aboral expression of *NvSix3/6* and *NvFz5/8*, whose opposing functions are essential for maintaining a correctly patterned aboral domain. These results imply that *Nematostella* embryos do not develop with a ‘default’ aboral/anterior identity in the absence of Nvβ-cat.

### Nvβ-cat determines oral and aboral domains

**Fig. 8.**
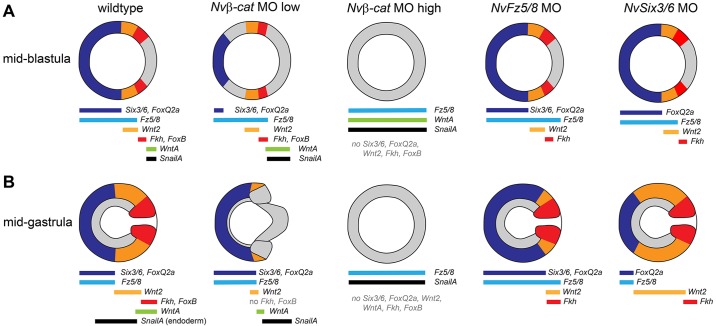
**Summary of the changes in gene expression in *Nvβ-c*at, *NvFz5/8* and *NvSix3/6* knockdown animals.** (A,B) Schematics of blastula (A) and gastrula (B) stage embryos, aboral pole to the left. *NvFoxQ2-*, *NvWnt2-* and *NvFkh-*expressing domains are indicated in dark blue, orange and red, respectively. *Nvβ-cat* morphants (low concentration) show at blastula stage a reduced aboral *NvSix3/6/NvFoxQ2a*-expressing territory and an expanded oral *NvWntA*/*NvSnailA*-expressing territory, and at gastrula stage an expanded endoderm-like *NvSnailA*-expressing territory. In *Nvβ-cat* morphants (high concentration), no polarized gene expression can be detected, but both oral (*NvWntA*, *NvSnailA*) and aboral (*NvFz5/8*) markers are expressed throughout the body. *NvFz5/8* and *NvSix3/6* morphants do not display alterations in gene expression at blastula stage. At gastrula stage, aboral markers are expanded towards the oral pole and oral markers are reduced in *NvFz5/8* morphants. Knockdown of *NvSix3/6* results in reduction of aboral and expansion of oral markers at gastrula stage.

Despite the role for Nvβ-cat activity in establishing both the oral and aboral ‘patterning modules’, our data, as well as previously published observations (Rottinger et al., 2012), do not support a simple model in which a gradient of Nvβ-cat activity determines the identity of different domains along the oral-aboral axis, with high levels determining oral and low levels determining aboral marker gene expression. At mid-blastula stage, *NvWntA* and *NvSnailA* are expressed in the oral-most domain whereas *NvFkh* and *NvFoxB* are expressed in a ring-like domain that excludes the oral-most area (Rottinger et al., 2012). We have shown that *NvFkh* and *NvFoxB* are downregulated by *Nvβ-cat* knockdown, whereas *NvWntA* and *NvSnailA* are upregulated (Fig. 3; summarized in Fig. 8). Furthermore, at blastula stage, the low dose of *Nvβ-cat* MO causes a consistent shift of the boundaries of expression domains towards the aboral pole and an expansion of the oral-most domain. We consider it unlikely that these effects are based on the role of β-catenin in cell adhesion, which we expect to be rather uniform along the oral-aboral axis at mid-blastula stage. Interestingly, embryos injected with the high dose of *Nvβ-cat* MO display no morphological or molecular polarity and do not gastrulate, whereas embryos injected with a lower dose show an expanded endoderm-like *NvSnailA*-expressing territory at gastrula stage (Figs 2 and 4). Although this suggests that different levels of Nvβ-cat are indeed involved in the specification of different territories, it also indicates that there is an unknown input into the *NvWntA-* and *NvSnailA-*positive territory at blastula stage, and that the size of this territory is negatively regulated by Nvβ-cat activity. The nature and regulation of this additional input remain to be studied.

The establishment of the aboral patterning module involves the localized activation (direct or indirect) of *NvSix3/6* in the aboral domain (Figs 3 and 4) and the repression of *NvFz5/8* in the oral domain (Fig. 6), both processes being dependent on Nvβ-cat. We currently cannot determine whether the activation of *NvSix3/6* is based on a direct role of Nvβ-cat within the aboral domain, or is a consequence of the function of Nvβ-cat in specifying oral identity. In the latter scenario, the oral domain would produce a signal that subsequently regulates aboral development. We observed that aboral isolates (generated by bisection at the eight-cell stage), which develop into epithelial spheres without polarity (Fritzenwanker et al., 2007; Lee et al., 2007), still express *NvSix3/6* (Fig. S8), indicating that *NvSix3/6* can be activated in the absence of signals from the oral domain. More refined experiments will be necessary to determine how Nvβ-cat regulates gene expression in the aboral territory.

A positive role of β-cat in the formation of apical/aboral regulatory networks has, to our knowledge, not been shown so far. In the sea urchin *Strongylocentrotus purpuratus* (Range et al., 2013; Yaguchi et al., 2008), the hemichordate *Saccoglossus kowalevskii* (Darras et al., 2011) and the hydrozoan *Clytia hemisphaerica* (Momose et al., 2008; Momose and Houliston, 2007) the activity of a blastoporal Wnt/β-catenin signaling center prevents the ectopic expression of the apical/aboral marker genes *Six3/6* and/or *FoxQ2.* Although antagonism of Wnt/β-catenin signaling and *Six3* is probably a common feature in the patterning of the anterior-posterior axis in bilaterians, our results suggest that in *Nematostella* this antagonism is only required for the maintenance and the refinement of oral-aboral patterning, but not for the initial formation of the oral and aboral territories.

### *Frizzled5/8* and *Six3/6* constitute an ancient aboral/anterior pole patterning system

Once the aboral expression domains of *NvSix3/6* and *NvFz5/8* are established, *NvSix3/6* positively regulates *NvFz5/8*, which in turn negatively regulates *NvSix3/6*, and the opposing functions of these two genes maintain the position of the boundary between oral and aboral territories (Fig. 6). Whether this process involves direct regulatory interactions between *NvSix3/6* and Wnt signaling components remains unclear. The effect of the *NvFz5/8* MO on aboral markers was detectable only from early gastrula stage onwards, when the *NvFz5/8*, *NvSix3/6* and *NvFoxQ2a* expression domains are similarly restricted to the aboral domain. Although this indicates that *NvFz5/8* might function within the aboral domain to inhibit *NvSix3/6* expression, it is also possible that *NvFz5/8* begins to regulate *NvSix3/6* as early as the blastula stage, when its expression extends further to the oral side than that of *NvSix3/6*, or that remaining NvFz5/8 protein in the oral domain is required to restrict *NvSix3/6* transcription to the aboral domain.

Opposing activities of *Frizzled5/8 *and* Six3/6* have also been described in the formation of the apical neuroectoderm in the sea urchin *Strongylocentrotus purpuratus* (Range et al., 2013; Wei et al., 2009) and overlapping anterior/apical expression of *Frizzled5/8* and *Six3/6* orthologs has been described in several protostomes (Marlow et al., 2014; Steinmetz et al., 2010; Beermann et al., 2011; Posnien et al., 2009) and deuterostomes (Lowe et al., 2003; Pani et al., 2012; Deardorff et al., 1998; Kim et al., 1998; Seo et al., 1998; Zhou et al., 2000). It thus seems likely that an interaction of *Six3/6* and *Frizzled5/8* in the anterior/aboral domain is an ancient feature of animal development, but that the role of Wnt/β-catenin signaling in its formation differs between species.

### Patterning within the aboral domain after gastrulation

Our data suggest that the role of *NvFz5/8* in the development of the apical organ after gastrulation is mainly mediated by its interaction with NvFGF signaling. At planula stage, the expression of *NvFGFa1* and *NvFGFa2* is no longer affected by *NvFz5/8* knockdown, and the expression of *NvFz5/8* itself becomes dependent on *NvFGFa1* (Fig. 7). *NvFz5/8* knockdown leads to the formation of an initially wider aboral domain. However, the FGF feedback loop at planula stage, and notably the repressive function of NvFGFa2 on NvFGFa1 signaling, is sufficient to prevent the formation of an expanded apical organ, leading paradoxically to the formation of a smaller apical organ. Only when the repressive function of *NvFGFa2* is blocked, the expansion of the aboral domain in *NvFz5/8* morphants is translated into a much expanded apical organ. The analyses at gastrula and planula stage suggest that the interaction between *NvFz5/8* and NvFGF signaling occurs only after gastrulation; however, we cannot exclude the possibility that earlier roles of these signaling molecules contribute to the phenotypes observed at planula stage.

### Upstream and downstream of *NvFz5/8*

NvWnt genes are expressed exclusively in the oral half of *Nematostella* (Kusserow et al., 2005; Lee et al., 2006), suggesting that the activity of *NvFz5/8* in restricting the aboral domain is regulated by signals emanating from the oral half. Consistent with this hypothesis, the effect of *NvFz5/8* knockdown became apparent only after the onset of expression of most ectodermal NvWnt genes at gastrula stage. We were able to phenocopy the *NvFz5/8* MO knockdown at gastrula stage by using a truncated *NvFz5/8* construct encoding the extracellular, ligand-binding domain and a partial transmembrane domain (Fig. S4). Presumably, this construct acts in a dominant-negative manner by competing with endogenous Frizzled receptors for the binding to Wnt ligands without being able to transduce a signal into the cell. Thus, the results obtained upon expression of this construct support the hypothesis that NvFz5/8 can bind Wnts and acts as a bona fide Wnt receptor. However, it remains unclear which Wnt ligand(s) might bind to NvFz5/8.

The lack of an effect on the initial establishment of the aboral territory argues that *NvFz5/8* does not activate β-catenin signaling at blastula stage. The role of *NvFz5/8* in regulating the size of the aboral domain resembles the role of *Frizzled5/8* in axial patterning in the sea urchins *Strongylocentrotus purpuratus* and *Paracentrotus lividus* (Croce et al., 2006; Range et al., 2013), which is mediated by JNK signaling. Application of a range of different concentrations of the JNK inhibitor SP600125 (which inhibits JNK activity in *Hydra*; Philipp et al., 2009) did not mimic the *NvFz5/8* knockdown phenotype (data not shown). Gene expression along the primary axis of *Nematostella* can be perturbed by pharmacological activation of the Wnt/β-catenin pathway throughout development and along the entire axis (Marlow et al., 2013; Trevino et al., 2011) and we were able to rescue the patterning defects of *NvFz5/8* morphants by exposing the animals to a low dose of AZ (1 μM) from mid-blastula stage onwards (Fig. S9). Although it is still possible that stabilization of Nvβ-cat in the oral domain indirectly affects the size of the aboral domain, we favor the hypothesis that the function of NvFz5/8 in patterning the ectoderm during gastrulation is mediated by Nvβ-catenin and not by JNK signaling.

In conclusion, we have identified an ancient mechanism for the patterning of the aboral/anterior embryonic territory depending on the activities of *Six3/6* and *Frizzled5/8.* In *Nematostella*, this system is established by a previously undescribed role for β-catenin in the development of both oral and aboral territories. These findings provide a deeper understanding of the evolutionary conservation and plasticity of the patterning of the animal primary body axis.

## MATERIALS AND METHODS

### *Nematostella vectensis* culture

*Nematostella* polyps were induced for spawning as described previously (Fritzenwanker and Technau, 2002). Embryos were cultured in one-third seawater (*Nematostella* medium) at 21°C.

### Overexpression constructs and mRNA injection

The open reading frame of *NvSix3/6* and codons 1-249 (extracellular plus first transmembrane domain) of *NvFz5/8* were each cloned into pENTR using the directional D-TOPO Cloning Kit (Invitrogen) and recombined into the destination vector pSPE3-RfA-Venus (Roure et al., 2007). For primer sequences see Table S1. *EGFP* mRNA was generated from pCS2+-EGFP. Messenger RNA was synthesized with the mMessage mMachine Kit (Ambion) and injected at 50 ng/µl (*NvSix3/6-venus*) and 90 ng/μl (dn*NvFz5/8-venus*) using dextran Alexa-568 as tracer. Only animals with clear Venus fluorescence at gastrula stage were used for analysis.

### Morpholino injection

Injections were carried out as described previously (Rentzsch et al., 2008). To compensate for slight developmental delay, all injected animals (control MO, gene-specific MO and mRNA) were fixed 2 h later than uninjected animals. The generic control MO was used for all images shown. Morpholino sequences are presented in Table S2. The phenotypes represented in the figures were observed in 60-90% of the injected animals unless stated otherwise. All experiments were performed with at least two replicates and a number of specimens per sample of >20.

### *In situ* hybridization and antibody staining

Colorimetric *in situ* hybridizations were performed as described previously (Rentzsch et al., 2006, 2008). Fixation for *in situ* hybridization, immunostaining (anti-acetylated tubulin antibody, Sigma T6793, batch 081M4760, 1:500; anti-mouse β-catenin, Sigma C2206, batch 062M4806, 1:500), Phalloidin and DAPI staining were performed as described by Leclere and Rentzsch (2014).

### AZ treatments

AZ (Calbiochem #191500) was dissolved to a concentration of 10 mM in DMSO; this stock was diluted in *Nematostella* medium to the final concentrations. Wild-type embryos were incubated from 2 hpf, injected embryos from 4 hpf onwards, except for *NvFz5/8* MO-injected animals, which were incubated from 14 hpf to 28 hpf.

### Quantitative RT-PCR

RT-qPCR was performed as described by Sinigaglia et al. (2013). Primers are listed in Table S1.

### Microscopy

Images were taken using a Nikon Eclipse E800, a Zeiss Axiophot or a Nikon AZ100M microscope and adjusted in Photoshop CS5. Confocal images were recorded on a Leica SP5 confocal microscope; confocal stacks were processed with the Leica software and adjusted in Photoshop.
